# Prognostic impact of AJCC response criteria for neoadjuvant chemotherapy in stage II/III breast cancer patients: breast cancer subtype analyses

**DOI:** 10.1186/s12885-016-2500-1

**Published:** 2016-07-21

**Authors:** Yaewon Yang, Seock-Ah Im, Bhumsuk Keam, Kyung‑Hun Lee, Tae‑Yong Kim, Koung Jin Suh, Han Suk Ryu, Hyeong-Gon Moon, Sae‑Won Han, Do‑Youn Oh, Wonshik Han, Tae‑You Kim, In Ae Park, Dong-Young Noh

**Affiliations:** Department of Internal Medicine, Seoul National University Hospital, Seoul National University College of Medicine, 101 Daehak-ro, Jongno-gu, Seoul 110-744 Korea; Cancer Research Institute, Seoul National University, Seoul, Korea; Department of Surgery, Seoul National University Hospital, Seoul National University College of Medicine, Seoul, Korea; Department of Pathology, Seoul National University Hospital, Seoul National University College of Medicine, Seoul, Korea

**Keywords:** Stage II or III, Breast neoplasm, Neoadjuvant chemotherapy, American Joint Committee on Cancer (AJCC) response, Relapse-free survival, Breast cancer subtype

## Abstract

**Background:**

Neoadjuvant chemotherapy (NAC) is a standard treatment for stage II/III breast cancer patients, and response to NAC is a useful prognostic marker. Since its introduction, 6–8 cycles of NAC has become the standard regimen to improve the outcome of these patients. The purpose of this study is to evaluate the prognostic impact of the American Joint Committee on Cancer (AJCC) response criteria and this tool’s usefulness in four different breast cancer subtypes.

**Methods:**

We conducted a retrospective cohort study of clinical stage II/III breast cancer patients who received NAC of more than 6 cycles. Response after NAC and the clinicopathological factors were reviewed. AJCC response criteria for NAC were adopted from the AJCC Manual, 7th edition: complete response (CR), partial response (PR), and no response (NR).

**Results:**

A total of 183 patients were enrolled; 22 (12.0 %), 123 (67.2 %), and 38 (20.8 %) patients showed CR, PR, and NR, respectively. The AJCC response was significantly associated with relapse-free survival (RFS) (*P* < 0.001), whereas pathologic CR (pCR), the current gold standard for response evaluation for NAC, was not (*P* = 0.140). AJCC response was a significant prognostic factor for RFS in all four breast cancer subtypes, namely luminal A (*P* = 0.006), luminal B (*P* = 0.001), HER-2 enriched (*P* = 0.039), and triple-negative breast cancer (*P* = 0.035).

**Conclusions:**

The AJCC response criteria represent a simple and easily reproducible tool for response evaluation of NAC patients and a useful clinical prognostic marker for RFS. These criteria also have a prognostic impact in all four breast cancer subtypes, including luminal A in which pCR has a limited role.

## Background

Breast cancer is the most common cancer in women worldwide, and 1.68 million cases are newly diagnosed annually [1]. It is the second most common cancer in females in South Korea, where 15,942 women were newly diagnosed in 2011 [2]. About 44 % of the newly diagnosed breast cancer patients are initially stage II or III, and neoadjuvant chemotherapy (NAC) or primary systemic therapy has become the standard treatment for this population [3, 4]. Response to NAC is known to be useful in prognostic and predictive aspects. Pathologic complete response (pCR) is the most useful surrogate marker for overall survival in the NAC setting [5–7]. However, despite its clinical usefulness, pCR alone has limitations in evaluating residual disease after NAC. Recent pooled analysis of neoadjuvant clinical trials revealed that pCR is not a surrogate end-point marker for survival in the overall population because response to NAC is heterogeneous among breast cancer subgroups [8]. Several methods have been devised to evaluate the response to NAC, one of which is the American Joint Committee on Cancer (AJCC) response criteria for NAC [9]. Keam et al. [10] validated AJCC response criteria for NAC in 398 patients who received 3 cycles of doxorubicin plus docetaxel, and found these criteria to be useful in evaluating the response to NAC as well as predicting survival after short-course NAC.

Since the middle of the last decade the importance of pCR achievement has been emphasized, and to obtain higher rates of pCR extended cycles of neoadjuvant chemotherapy have been introduced [11, 12]. Six to eight cycles of NAC has recently become the standard treatment in clinical practice. In this study, we evaluated the clinical impact of AJCC response criteria in patients undergoing six or more cycles of NAC. In addition, we evaluated the clinical usefulness and prognostic value of the AJCC criteria in four different breast cancer subgroups [13].

## Methods

### Study population and treatment

We conducted a retrospective cohort study of the patients who received NAC in Seoul National University Hospital. Between January 2009 and December 2010, all stage II/III breast cancer patients receiving NAC were screened.

Detailed eligibility criteria were as follows: (1) pathologically confirmed breast cancer by core needle biopsy; (2) clinical stage II or III; (3) presence of objective measurable lesion by Response Evaluation Criteria In Solid Tumors (RECIST) version 1.1 [14]; (4) Eastern Cooperative Oncology Group performance status 0–2; (5) previously untreated; (6) cycles of neoadjuvant chemotherapy of 6 or more. Initial evaluation included physical examination, mammography, breast ultrasonography, chest computed tomography (CT), bone scan, and breast magnetic resonance imaging (MRI). Initial tumor size was measured by MRI. Initial nodal staging was determined by physical examination and CT. After completing six or more cycles of neoadjuvant chemotherapy before definitive surgery, the patients were re-examined for response evaluation. Thereafter, the patients received curative surgery followed by adjuvant chemotherapy according to the physician’s decision, considering response to NAC and final pathologic stage [15]. Patients received additional adjuvant radiation therapy [16], trastuzumab [17, 18], and hormonal therapy [19], if indicated.

The study protocol was reviewed and approved by the Institutional Review Board at the Seoul National University Hospital (H-0510-506-159). Recommendations of the Declaration of Helsinki for biomedical research involving human subjects were also followed.

### Response evaluation

For evaluation of radiologic response, we obtained ultrasonography and MRI for primary breast cancer and chest CT for lymph node evaluation before and after NAC. The radiologic response was evaluated by RECIST criteria version 1.1 [14]. The initial clinical and post-NAC pathologic staging was based on the AJCC Cancer Staging Manual, 7th edition. The details of AJCC response criteria for NAC were as follows [9].

1. Complete response (CR) is defined as the absence of invasive carcinoma in the breast and lymph nodes. Residual in situ cancer, in the absence of invasive disease, constitutes a CR. Patients with isolated tumor foci in lymph nodes are not classified as having a CR.
2. Partial response (PR) is defined as a decrease in either or both T or N stage compared to the pretreatment T or N, and no increase in either T or N. After chemotherapy, one should use the method that most clearly defined tumor dimensions at the baseline for this comparison, although pre-chemotherapy pT cannot be measured.
3. No response (NR) is defined as no apparent change in either the T or N categories compared to the clinical pretreatment assignments, or increase in either the T or N categories at the time of pathologic evaluation.

Pathologic complete response (pCR) is defined as complete disappearance of invasive carcinoma, in both the breast and the axillary lymph nodes, after NAC. Residual ductal carcinoma in situ (DCIS) was included in the pCR category.

### Clinicopathological examination

The clinical characteristics (age at diagnosis, date of diagnosis, date, cycles and regimen of neoadjuvant chemotherapy, date of surgery, adjuvant therapy, date of last visit, date of relapse) and the laboratory test results (follicle-stimulating hormone, luteinizing hormone, and estradiol levels at diagnosis for determination of menopausal status [20]) were obtained by retrospective review of electronic medical records. We performed immunohistochemistry (IHC) using tissues obtained at diagnosis. Estrogen receptor (ER), progesterone receptor (PR), human epidermal growth factor receptor 2 (HER2), p53, Bcl-2, epidermal growth factor receptor (EGFR), and Ki-67 expression were evaluated. IHC was performed as previously described in our center’s study series [21–24]. In the case of HER2 IHC 2+, fluorescence in situ hybridization (FISH) was performed to determine HER2 positivity. Positivity thresholds for classification were ER ≥ 1 %; PR ≥ 1 % [25]; HER2 = IHC 3+ (>10 % invasive tumor cells with intense and circumferential membrane staining) and/or FISH positive (HER2/CEP17 ratio ≥ 2.2) [26, 27]; and p53 ≥ 25 % [22, 28]. The Ki-67 threshold of high (≥14 %) was based on work by Cheang et al., in which 14 % best discriminated between luminal-A and luminal-B tumors [29].

### Breast cancer subtypes

Breast cancer is further classified into several groups according to their molecular alteration, cellular composition, and clinical outcome. Tumor classification is useful in determining and predicting response to treatment as well as providing prognostic information. In this study, we classified breast cancer patients into four subgroups, namely luminal A (LA), luminal B (LB), HER2 enriched (HER2), and triple-negative breast cancer (TNBC), these definitions being mainly adopted from the 2011 St Gallen Consensus Panel [30]. Definitions of each subgroup are as follows.

1. LA (highly endocrine responsive): ER positive, PR positive, HER2 negative, and Ki-67 low. The few ER-negative/PR-positive cases were considered ER-positive/PR-positive.
2. LB (moderately endocrine responsive): ER positive and PR negative independent of other parameters, or ER positive, PR positive and at least one of grade 3, HER2 positive, and/or Ki-67 high.
3. HER2: ER negative, PR negative, and HER2 positive.
4. TNBC: ER negative, PR negative, and HER2 negative regardless of the expression of EGFR and basal cytokeratins.

Although there is some controversy surrounding endocrine therapy for patients with low ER-expressing tumors (1–10 %, weakly positive) regarding the benefit of tamoxifen and other endocrine therapies on survival and their relatively low toxicities, the American Society of Clinical Oncology (ASCO) and the College of American Pathologists (CAP) Panel recommended that ER and PR assays be considered positive if there are at least 1 % positive tumor nuclei in the sample [25, 31, 32]. The recent National Comprehensive Cancer Network guideline for breast cancer also adopted the ASCO/CAP recommendation. We adopted the ASCO/CAP guideline to determine the hormone receptor-positive tumors.

### Statistical analysis

Relapse-free survival (RFS) was determined as the interval between the initiation of neoadjuvant chemotherapy and the date when disease relapse or progression was first documented, or the date of death from any cause. Local, regional, and distant relapse were all included in disease relapse, and contralateral breast cancer was not regarded as relapse. The Kaplan–Meier product limit method and the Cox proportional hazards regression model were used for survival analyses. Log-rank tests were used to compare RFS between different subgroups. Differences between breast cancer subtypes with regard to clinicopathologic characteristics were examined using 1-way analysis of variance (ANOVA) for the continuous variables (age, pre- and post-NAC tumor size), and *χ*^2^ tests for the remaining variables. All statistical tests were two-sided, with the level of significance established at *P* < 0.05. All statistical analyses were carried out using SPSS version 21.0 (SPSS, Chicago, IL, USA).

## Results

### Patients and treatment

During the study period, 249 stage II/III breast cancer patients received NAC in Seoul National University Hospital. Sixty-six patients were excluded because they received less than six cycles of NAC, and finally 183 patients of median age 46 (range 25–71) years were enrolled and evaluated. The median follow-up duration was 38.0 (range 9–53) months. At the data cut-off point in June 2013, 41 patients (22.4 %) had developed recurrent disease. The median RFS was not reached at the time. One hundred and fifty-three patients (83.6 %) were stage III. One hundred and nine (59.6 %) were premenopausal, and 106 (57.9 %) had hormone receptor-positive tumors. The majority of the patients received both anthracycline- and taxane-containing NAC. A total of 128 (69.9 %) received a concurrent anthracycline and taxane regimen, and 47 (25.7 %) received sequential anthracycline and taxane. Ten patients (32.3 % of HER2-positive patients) received a HER2-targeted agent (trastuzumab or trastuzumab emtansine)-containing NAC regimen. The baseline characteristics of the 183 patients are summarized in Table 1.

**Table 1:** Baseline characteristics

Variables	Number of patients (*N* = 183)	
	No	%
Age, median (range)	46 (25–71)	
Histology		
Invasive ductal carcinoma	167	91.3
Premenopausal/Postmenopausal	109/74	59.6/40.4
Regimen of Neoadjuvant Chemotherapy		
Concurrent anthracycline + taxane	128	69.9
Sequential anthracycline + taxane	47	25.7
HER-2 directed agent containing regimen	10	5.5
Doxorubicin plus cyclophosphamide	1	0.5
Type of Surgery		
Breast conserving surgery	104	56.8
Mastectomy	79	43.2
Initial Clinical Stage		
IIA	3	1.6
IIB	27	14.8
IIIA	94	51.4
IIIB	26	14.2
IIIC	33	18.0
Hormone receptor and HER2 expression status		
Hormone receptor (+)	106	57.9
HER2 (+)	58	31.7
Pathologic Stage		
yp0	22	12.0
ypIA	41	22.4
ypIIA	42	23.0
ypIIB	22	12.0
ypIIIA	38	20.8
ypIIIB	2	1.1
ypIIIC	16	8.7
Adjuvant therapy		
Radiation therapy	158	86.3
Chemotherapy	62	33.9
Trastuzumab	55	94.8 % of patients with HER2 positive tumor
Hormonal therapy	103	97.1 % of patients with HR positive tumor

*HER2* human epidermal growth factor receptor 2, *TNBC* triple negative breast cancer

### Response to the neoadjuvant chemotherapy and relapse-free survival

**Fig. 1:**
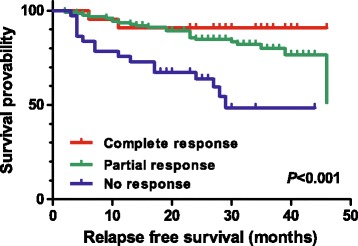
Relapse-free survival probability according to the American Joint Committee on Cancer (AJCC) response, complete response, partial response, and no-response groups

**Fig. 2:**
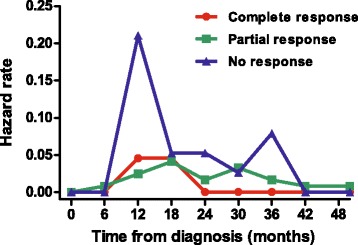
Hazard rates of relapse of breast cancer patients after diagnosis according to the AJCC response criteria

**Fig. 3:**
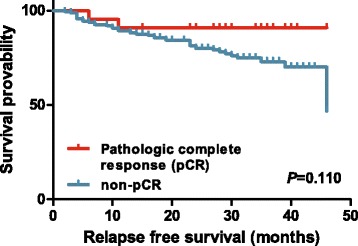
Relapse-free survival probability according to the pathologic complete response

The results of the response evaluation after NAC according to the AJCC response criteria are shown in Table 2. Among 183 patients there were 22 with CR (12.0 %), 123 with PR (67.2 %), and 38 with NR (20.8 %). The 3-year RFS rates were 90.9 % in CR, 79.9 % in PR, and 48.5 % in NR patients (Fig. 1, log-rank *P* < 0.001). AJCC response was significantly associated with RFS (the hazard ratio (HR) for relapse of CR/PR group vs. NR group was 0.269, with 95 % confidence interval (CI) of 0.141–0.513, *P* < 0.001). Figure 2 shows the hazard rate of relapse at the specific time from diagnosis (months) according to the three AJCC response groups. In the NR group, about 27 % of patients relapsed within 1 year and 52 % relapsed within the first 3 years from diagnosis, even during the adjuvant chemotherapy, radiotherapy, or hormone therapy. The AJCC response CR and PR groups showed similar prognosis and no statistical difference of RFS probability (HR of PR group 2.067, 95 % CI 0.485–8.803, *P* = 0.326), but the PR and NR groups showed a significant difference in RFS probability (HR of NR group 3.665, 95 % CI 1.879–7.146, *P* < 0.001). After adjusting for potential prognostic factors, the AJCC response was independently associated with RFS (*P* = 0.004, Table 3), as well as the traditional prognostic factors such as pathologic stage and hormone receptor positivity. The pCR was not a significant predictor of RFS (Fig. 3, log-rank *P* = 0.110), despite the Kaplan–Meier curve showing a tendency for survival difference between the two groups.

**Table 2:** AJCC response after neoadjuvant chemotherapy

AJCC response	Number of patients (*N* = 183)	
	Number	Percent (%)
CR	22	12.0
PR	123	67.2
NR	38	20.8

*CR* complete response, *PR* partial response, *NR* no response

**Table 3:** Univariate and multivariate analyses for relapse free survival

Variables	Univariate analysis			Multivariate analysis		
	HR	95 % CI	*P*	HR	95 % CI	*P*
Age (≤median)	0.685	0.364–1.287	0.240		-	
Menopausal status (Premenopausal)	0.846	0.449–1.594	0.605		-	
pCR	0.342	0.082–1.420	0.140		-	
AJCC response (CR plus PR)	0.309	0.172–0.556	0.000	0.374	0.192–0.728	0.004
Surgery (BCS)	0.428	0.228–0.803	0.008	0.475	0.252–0.894	0.021
Pathologic Stage (yp0,I)	0.235	0.092–0.600	0.002	0.267	0.094–0.756	0.013
Hormone receptor positive	0.447	0.236–0.846	0.013	0.402	0.198–0.818	0.012
HER2 positive	1.059	0.761–1.474	0.734		-	
TNBC	1.038	0.056–2.131	0.919		-	
Subtype (Luminal A)	0.379	0.145–0.985	0.047	0.314	0.103–0.962	0.043

*pCR* pathologic complete response, *CR* complete response, *PR* partial response, *BCS* breast conserving surgery, *HER2* human epidermal growth factor receptor 2, *TNBC* triple negative breast cancer, *HR* hazard ratio, *95 % CI* 95 % confidence interval, *P* p-value

### Breast cancer subgroup analysis

**Fig. 4:**
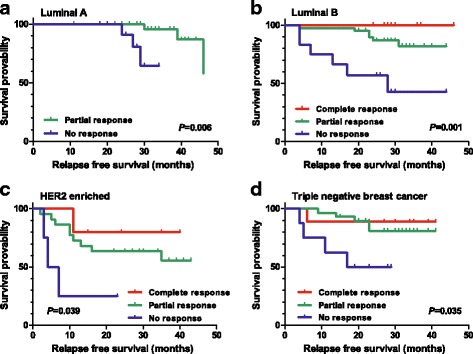
Relapse-free survival probability according to the AJCC response in each breast cancer subgroup: **a** luminal A type, **b** luminal B type, **c** HER2-enriched type, and **d** triple-negative breast cancer type

We divided the patients into four subgroups (LA, LB, HER2, and TNBC) as previously described, comprising 44 (24.1 %), 62 (33.9 %), 31 (16.9 %), and 46 (25.1 %) patients, respectively. The rate of pCR was higher in TNBC (19.6 %), HER2 (16.1 %), and LB (12.9 %) groups than in the LA (0 %) group. The rate of AJCC response of CR plus PR was also higher in the TNBC (82.6 %), HER2 (87.1 %), and LB (79.0 %) groups than in the LA (70.5 %) group. The response to the NAC according to the AJCC criteria was a significant prognostic factor for RFS in all four subgroups: LA (*P* = 0.006), LB (*P* = 0.001), HER2 (*P* = 0.039), and TNBC (*P* = 0.035) (Fig. 4, log-rank test). In all subgroups, CR and PR patients showed a similar hazard rate of relapse: HR of PR patients was not assessable (no CR in LA group), 26.953 (*P* = 0.618), 2.690 (*P* = 0.345), and 1.613 (*P* = 0.663) in LA, LB, HER2, and TNBC groups, respectively. Interestingly, NR patients showed a significantly higher risk of relapse than PR patients in all the four breast cancer subgroups: HR of NR patients was 12.898 (95 % CI 1.285–129.437, *P* = 0.030), 4.224 (95 % CI 1.088–16.394, *P* = 0.037), 5.044 (95 % CI 1.555–16.366, *P* = 0.007), and 4.206 (95 % CI 1.121–15.786, *P* = 0.033) in the LA, LB, HER2, and TNBC groups, respectively. On the contrary, pCR was not significantly associated with RFS in any subgroups (Table 4). Pathologic stage was significantly associated with RFS in HER2 and TNBC patients, but not in the LA and LB groups. Table 4 shows the prognostic factors for RFS in each breast cancer subgroup by Cox regression.

**Table 4:** Prognostic factors according to subgroup analysis

Variables	Luminal A			Luminal B			HER2			TNBC		
	HR	95 % CI	P	HR	95 % CI	P	HR	95 % CI	P	HR	95 % CI	P
Age												
≤ median	0.477	0.078–2.907	0.422	0.646	0.207–2.019	0.453	0.616	0.188–2.016	0.423	0.597	0.168–2.115	0.424
> median	1			1			1			1		
Menopausal status												
Premenopausal	0.092	0.009–0.906	0.041	0.875	0.282–2.727	0.818	0.482	0.132–1.754	0.268	0.741	0.214–2.558	0.635
Postmenopausal	1			1			1			1		
pCR		-		0.039	0.000–46.767	0.370	0.360	0.047–2.773	0.327	0.420	0.053–3.323	0.411
vs non-pCR				1			1			1		
AJCC response												
CR plus PR	0.078	0.008–0.778	0.030	0.157	0.050–0.499	0.002	0.221	0.058–0.838	0.026	0.219	0.061–0.783	0.020
NR	1			1			1			1		
Pathologic Stage												
yp0,I	0.517	0.247–1.081	0.080	0.160	0.021–1.238	0.079	0.162	0.036–0.732	0.018	0.182	0.038–0.861	0.032
ypII, III, IV	1			1			1			1		
Surgery												
BCS	0.414	0.069–2.493	0.336	0.329	0.101–1.071	0.065	0.296	0.081–1.081	0.065	0.930	0.240–3.660	0.917
Mastectomy	1			1			1			1		

*pCR* pathologic complete response, *CR* complete response, *PR* partial response, *NR* no response, *BCS* breast conserving surgery, *HER2* human epidermal growth factor receptor 2, *TNBC* triple negative breast cancer, *HR* hazard ratio, *95 % CI* 95 % confidence interval, *P* p-value

## Discussion

Response to the neoadjuvant chemotherapy in stage II or stage III breast cancer has a prognostic impact on RFS and overall survival [33–35]. pCR is the current standard criterion for evaluation of the response after NAC [5–7]. Addition of preoperative taxanes to doxorubicin and cyclophosphamide (AC) increased the proportion of patients with pCR compared with preoperative AC alone (26 % vs. 13 %, respectively; *P* < 0.001) [11, 36]. Following publication of the results of NSABP B-27 together with B-18 and several other studies, extended NAC to obtain a higher pCR rate became the standard treatment in clinical practice [11, 12, 36, 37]. Despite its clinical usefulness, pCR has some limitations as a prognostic and predictive marker, and several groups proposed new methods for grouping post-NAC patients to evaluate the response to NAC [35, 38, 39], one of which is the AJCC response criteria for NAC. A previous study by Keam et al. [10] showed that the AJCC response criteria for NAC correlate well with radiologic response criteria and have a prognostic value for both RFS and overall survival in patients with three cycles of concurrent doxorubicin plus docetaxel neoadjuvant chemotherapy. CR, PR, and NR rates were 9.8, 59.3, and 30.7 %, 5-year RFS 89.6, 74.1, and 62.6 % (*P* = 0.002), and 5-year overall survival 97.4, 88.6, and 78.3 % (*P* = 0.012), respectively [10]. In the current study, we demonstrated that the AJCC response criteria represent a useful prognostic factor for RFS in patients undergoing 6 or more cycles of NAC. The rates of CR and PR are significantly higher (12 and 67.2 %, respectively) than those from 3 cycles of NAC in the previous study [10]. Because the follow-up duration is short, the prognostic impact of AJCC response with extended NAC on the overall survival has not yet been obtained.

Previous studies reported different rates of pCR after NAC between breast cancer subgroups, and suggested the clinical usefulness of pCR as a surrogate marker of survival is different in each breast cancer subtype [13]. According to von Minckwitz et al., pCR is a suitable surrogate end point for patients with LB/HER2-negative, HER2-positive (non-luminal), and triple-negative disease but not for those with LB/HER2-positive or LA tumors [13]. LA is a slowly proliferating tumor type, whose response to NAC is not as good as that in highly proliferating tumor types. Given these heterogeneous responses to NAC, a recent meta-analysis revealed that pCR alone is not sufficient as a surrogate end point for event-free survival and overall survival in the general breast cancer population [8]. In fact, no patients achieved pCR in the LA group in our current study. Furthermore, in contrast to previous studies, pCR was not associated with RFS in any of our subgroups. This might result from a short follow-up period and a lack of sufficient events (relapse) for obtaining statistical power. By contrast, our analyses of the four breast cancer subtypes demonstrated that AJCC response after NAC was a significant prognostic marker for RFS in all four breast cancer subgroups, even in LA patients.

There was no significant difference in RFS between pCR and non-pCR groups (*P* = 0.110, log-rank test). The AJCC response in CR and PR patients showed a similar prognosis, but PR and NR groups showed a significant difference in RFS probability. We also compared the RFS of CR + PR groups with that of the NR group using AJCC response criteria. The RFS of CR + PR patients was significantly longer than that in the NR group (median RFS not reached vs. 29 months, *P* < 0.001, log-rank test). The RFS probability difference between the CR and PR groups was not significantly different, although the HR of PR patients was higher, at 2.067 (95 % CI 0.485–8.803, *P* = 0.326). This might result from the small sample size and, thus, insufficient events (sample size 145 and 24 events at the data cut-off). The AJCC response criteria may thus represent a simple and easily applicable tool to evaluate residual disease and a new surrogate end point in neoadjuvant trials. Further follow-up is needed to confirm the prognostic impact of the AJCC response criteria and pCR in each breast cancer subgroup.

We further analyzed the LA patients. In our hospital’s NAC cohort, we were able to obtain the clinicopathologic and survival data of all the LA patients, regardless of the number of NAC cycles (<6 or ≥6 cycles). For the LA patients, pCR was achieved in neither group. The AJCC response rate (CR + PR portion) was higher in the extended-NAC group than in the short-course NAC group (70.5 % vs. 56.3 %), although statistically not significant (*P* = 0.302). This trend is consistent with the previous report by Moon et al. [40], which showed that continuous tumor shrinkage occurred in their ER-positive tumor group during extended NAC, while tumor shrinkage mainly occurred in the early period of NAC in the ER-negative group. By Cox regression of prognostic factors on RFS in the LA subgroup, premenopausal patients showed a significantly lower risk of relapse than the postmenopausal patients (HR 0.092, 95 % CI 0.009–0.906, *P* = 0.041; Table 4). This benefit might result from the additional secondary ovarian function suppression effect of chemotherapeutic agents in premenopausal patients. All things considered, in younger LA patients in premenopausal status an extended-NAC strategy might be more beneficial.

From the hazard rate of RFS of the patients according to AJCC responses (Fig. 2), we discerned several clinical implications. In the NR group 27, 41, and 52 % of the patients relapsed during first 1, 2, and 3 years after diagnosis, respectively. For these patients, thorough physical examination and work-up for locoregional and/or distant metastases should be performed even during adjuvant therapies. From these data, we may suggest the necessity of further adjuvant chemotherapy even after the use of both an anthracycline- and taxane-containing NAC regimen and the selection of high-risk patients who require adjuvant chemotherapy. Ongoing adjuvant clinical trials (JBCRG04 (CREATE-X), NCT01864746 (PENELOPE-B)) are targeting high-risk patients with residual diseases after neoadjuvant chemotherapy. The recent results of the CREATE-X trial revealed that treatment with adjuvant capecitabine increased disease-free survival for patients with HER2-negative breast cancer who had residual disease after neoadjuvant chemotherapy [41]. The data also showed a tendency toward improving overall survival, albeit statistically insignificant, which might derive from the short follow-up period.

Our study has some limitations. First, because of the retrospective design of the study, the probability existed of selecting patients with a good response to NAC (whose cancer did not progress during NAC) while excluding patients with early progression or non-response. The investigation of those who received a short course of NAC (<6 cycles, total *n* = 66) provided some clues. They encompassed both groups who were initially scheduled to receive a short course of chemotherapy and who progressed during NAC. Although it was difficult to distinguish the two groups by retrospective medical record review, there was only one patient with definite clinical disease progression during NAC who received mastectomy after three cycles of NAC. According to the radiologic response criteria, there was no difference in the portion of patients with progressive disease (4.5 % vs. 4.4 %, <6 cycles vs. ≥6 cycles of NAC). Therefore, the selection bias is expected to be minimal in this study. Second, the follow-up duration in this study is short, so the prognostic impact of the AJCC response criteria on overall survival for a long course of NAC could not be demonstrated. Third, the sample sizes of the each breast cancer subgroup are rather small, so the prognostic impact of the AJCC response criteria in each breast cancer subtype should be investigated in a larger population in subsequent studies.

Despite these limitations, this is the first report to demonstrate the clinical usefulness of AJCC response criteria in patients undergoing six or more cycles of NAC with neoadjuvant chemotherapy regimens used in clinical practice. Furthermore, our analyses demonstrated that the AJCC response criteria represented a significant prognostic marker for RFS in all four breast cancer subgroups, including the LA subgroup in which pCR has a limited role. The AJCC response criteria serve as a simple and easily reproducible tool for response evaluation in breast cancer patients in the NAC setting in comparison with the classically used Residual Cancer Burden measurement method or Miller-Payne grading system [35, 38]. The AJCC response criteria could help overcome the limitations of pCR, as they may be valid in all breast cancer subgroups and be helpful in selecting those high-risk patients who need further adjuvant treatment. In addition, we performed a pre- and post-NAC paired imaging study (breast MRI or chest CT with breast ultrasonography) for accurate clinical staging, examination of radiologic response, and evaluation of AJCC response. Further follow-up is needed to establish the potential prognostic role of the AJCC response criteria and other clinicopathologic markers of overall survival in the NAC setting of six to eight cycles.

## Conclusions

The AJCC response criteria represent a simple and easily reproducible clinical tool for predicting RFS in patients with stage II/III breast cancer undergoing six or more cycles of neoadjuvant chemotherapy. It has a prognostic impact in all four breast cancer subtypes, including the LA group in which pCR has a limited role. The AJCC response to NAC could also be a useful tool for selecting high-risk patients who need further adjuvant chemotherapy and more thorough examination for relapse, together with classical prognostic markers such as pathologic stage and breast cancer subtypes.

## Abbreviations

AJCC, American Joint Committee on Cancer; CI, confidence interval; CR, complete response; ER, estrogen receptor; HER2, human epidermal growth factor receptor 2; HR, hazard ratio; IHC, immunohistochemistry; LA, luminal A; LB, luminal B; NAC, neoadjuvant chemotherapy; NR, no response; pCR, pathologic CR; PR, partial response; PR, progesterone receptor; RFS, relapse-free survival; TNBC, triple-negative breast cancer
